# Development and validation of an AI-driven blood glucose prediction system for pilots based on continuous glucose monitoring

**DOI:** 10.3389/fphys.2026.1902975

**Published:** 2026-08-14

**Authors:** Dalong Guo, Guangyun Wang, Dexin Kong, Zhen Tian, Baosen Tan, Yufei Qin, Zhaoting Yang, Yubin Zhou, Haocheng Yin, Yimeng Zhang

**Affiliations:** 1 Air Force Medical Center, Air Force Medical University, Beijing, China; 2 Guangzhou Institute of Technology, Xidian University, Guangzhou, China; 3 School of Microelectronics, Key Laboratory of Wide Band-Gap Semiconductor Materials and Devices, Xidian University, Xi’an, China

**Keywords:** aviation medicine, aviation pilot, continuous glucose monitoring (CGM), glucose prediction, multimodal fusion, transformer

## Abstract

During flight, pilots are exposed to unique environmental factors such as low air pressure, acceleration, and psychological stress, which can lead to altered glucose metabolism that poses a threat to flight safety. Traditional fingerstick blood glucose monitoring has drawbacks such as the need for intermittent readings, time-consuming procedures, and passive monitoring. Conventional continuous glucose monitoring (CGM) can only display historical and real-time glucose data and lacks predictive capabilities. In the present study, an artificial intelligence (AI)-driven blood glucose prediction system for pilots based on CGM and a multimodal transformer was developed with the aim of achieving proactive early warning of blood glucose risks among pilots. Sixty healthy male volunteers aged 25–50 years were recruited. Subjects completed 180 simulated flight experiments, during which multimodal data such as CGM glucose level, heart rate, blood oxygen saturation, and physical activity levels, were simultaneously collected to establish a scenario-specific dataset in aviation. A multimodal transformer model was developed to predict future blood glucose levels at 15, 30, and 60 min, and its performance was compared with baseline models such as linear extrapolation, long short-term memory (LSTM), and gated recurrent unit (GRU).The results showed that the mean absolute percentage error (MAPE) of the model for the prediction of blood glucose at 30 min was 7.2%, and the root mean square error (RMSE) was 9.4 mg/dL, which indicated a significantly superior prediction performance compared with the baseline models. For hypoglycemia event prediction, sensitivity was 92.5%, specificity was 89.7%, and the area under the receiver operating characteristic (ROC) curve was 0.94. A Clarke error grid analysis revealed that 98.7% of the predicted points fell within the clinically accurate region, which could potentially provide pilots with approximately 32 min of lead time for intervention. This system proved capable of accurately predicting blood glucose fluctuations during flight. Therefore, it may aid in shifting the metabolic health management of pilots from passive monitoring to proactive prevention, and serve as a novel tool for assuring aviation medical safety.

## 1 Introduction

Commercial airline pilots are constantly exposed to multiple unique physiological stresses in flight, including low air pressure, mild hypoxia, acceleration, flight-related stress, and altered sleep patterns. Even pilots with normal blood glucose metabolism can experience rapid drops in blood glucose levels, a tendency toward hypoglycemia, or even overt hypoglycemic events under the combined effects of a high-altitude environment and flight-related stress (Louis and Punjabi, 2009; Kelly et al., 2010). Hypoglycemia can directly cause confusion, slowed reactions, and impaired judgment in pilots. In severe cases, it can even induce in-flight incapacitation (Schoenborn et al., 2019). A close physiological association has also been found between blood glucose fluctuations and flight fatigue. Rapid drops in blood glucose or persistent hypoglycemia can lead to an insufficient energy supply to brain tissue, directly causing central nervous system fatigue, decreased attention, slowed reaction times, and impairment of working memory (Johnson et al., 2007). Compared with the general population, pilots operate in high-risk, high-responsibility environments where hypoglycemia-induced operational errors can have catastrophic consequences. Under normal ground conditions, clinically significant hypoglycemia is rare in individuals with intact glucose regulatory function. However, accumulating evidence indicates that the combined stress of hypobaric hypoxia, high cognitive workload, irregular meal timing, and sleep disruption during flight can disrupt glucose homeostasis in metabolically healthy adults: acute altitude hypoxia has been shown to suppress fasting plasma glucose and delay the counter-regulatory hormonal response to glucose decline, increasing susceptibility to transient hypoglycemia during long-haul cruise phases (Louis and Punjabi, 2009; Kelly et al., 2010). Therefore, monitoring blood glucose fluctuations and providing early warnings of hypoglycemia in the general pilot population are of critical value to aviation safety.

Traditionally, blood glucose monitoring in pilots has been based primarily on fingerstick blood tests before and during flights. Although this point-based monitoring approach meets basic safety requirements, it has several inherent limitations. First, fingerstick blood tests only reflect the blood glucose level at the time of sampling and cannot capture dynamic changes during flight. Second, the procedure is relatively time-consuming. A single fingerstick blood test requires an average of 47 seconds to complete. During a 5-h flight, pilots are required to perform at least seven blood glucose tests in accordance with the ARA.MED.330 protocol of the UK Civil Aviation Authority, with a total testing time of more than 5 min (Fan et al., 2024). For pilots who need to attend to their instruments and the external environment, the distraction caused by this procedure cannot be overlooked. In addition, frequent fingerstick blood tests lead to additional psychological burden and discomfort for pilots.

The advent of continuous glucose monitoring (CGM) technology offers a new approach to address the challenges described above. CGM involves real-time monitoring of interstitial fluid glucose levels through a subcutaneous sensor, which automatically records data every 5 to 15 min to generate a continuous fluctuation curve of blood glucose. In recent years, several research teams have conducted systematic assessments of the precision and reliability of CGM in aviation environments. In a simulated flight study conducted in a low-pressure chamber, Fan et al. found that the correlation between CGM and fingerstick blood glucose monitoring reached 0.96 under conditions of flight-related changes in air pressure, and the mean absolute relative difference (MARD) showed minimal differences between flight and ground conditions (Fan et al., 2025). A real-world flight study by Meçani et al. further confirmed that standard CGM monitors such as Dexcom G7 and Abbott FreeStyle Libre 3 demonstrated stable accuracy throughout flight, with the overall MARD for the devices being 9.5% and 9.6%, respectively (Meçani et al., 2026). CGM also achieved significantly higher satisfaction levels in terms of pilot user experience, with all interviewed pilots expressing high confidence in the CGM results. In contrast, only two-thirds of those using traditional fingerstick testing reported the same level of confidence (Fan et al., 2025).

Despite its advantages, CGM provides only current and past blood glucose data and is incapable of predicting future trends. Its “post-event monitoring” mode means that it is essentially passive, with pilots only able to identify risks and adopt intervention measures when the blood glucose level has already deviated from the normal range. The key to achieving true proactive prevention lies in the shift from “monitoring” to “prediction”.

Rapid advances in artificial intelligence (AI) technology in the field of blood glucose prediction have provided the potential to make such a leap. Recently, researchers have explored a variety of predictive models, ranging from traditional machine learning to deep learning. Traditional approaches involve the widespread application of algorithms such as XGBoost and random forest to predict blood glucose, with certain studies reporting F1 scores >96% (Kozinetz et al., 2024). Prediction performance has been improved further with the use of deep learning models: long short-term memory (LSTM) networks have become commonly used baseline models in this field due to their superior ability to process temporal data. Previous research has shown that LSTM can achieve a mean absolute percentage error (MAPE) of 11.1% for a 30-min prediction horizon (Zhu et al., 2025). The transformer architecture, with its self-attention mechanism, has demonstrated unique advantages in capturing long-range dependencies. Multiple studies have confirmed that it outperforms traditional recurrent neural network (RNN) models in blood glucose prediction tasks (Rodriguez-Almeida et al., 2025). Recent work has demonstrated the feasibility of non-invasive glucose estimation using peripheral physiological signals, with a study developing and validating a non-invasive glucose monitor based on oxygen saturation and heart rate signals under ground static conditions (Mahmood et al., 2025). However, these approaches have not been tested in aviation hypoxic stress environments, nor have they quantified the independent predictive contribution of each physiological modality. Of particular interest is GluFormer, a CGM data-based generative foundation model presented in a study published in *Nature* in 2026 (Lutsker et al., 2026). This model was pre-trained in a self-supervised manner on a massive dataset comprising more than 10 million blood glucose measurements. In addition to accurately predicting short-term blood glucose fluctuations, it was able to extract features with long-term prognostic value from a single CGM curve. In a cohort that was followed for 11 years, GluFormer showed superior performance over traditional hemoglobin A1C (HbA1c) indicators in identifying future risks of diabetes mellitus and cardiovascular death: 66% of new diabetes cases and 69% of cardiovascular deaths occurred in the quartile with the highest risk (Lutsker et al., 2026) This finding revealed that CGM data contained far more information than just blood glucose levels, thereby providing key insights for developing a pilot-specific metabolic risk assessment system.

Applications of large language models (LLMs) in the field of blood glucose prediction have also gradually emerged. A recent study systematically compared the performance of traditional machine learning, deep learning, and LLMs in blood glucose prediction tasks among patients with type 1 diabetes mellitus. The study found that GPT-4o achieved the highest accuracy for the 30-min and 60-min prediction horizons, whereas LLaMA-3.2 1B exhibited the best performance in the 90-min long-term blood glucose prediction task. These results confirmed the potential advantages of LLMs in modeling the temporal blood glucose trajectory and performing long-term prediction. More importantly, LLMs can explain the basis for their predictions in natural language. To some extent, this alleviates trust-related clinical concerns caused by the “black box” nature of deep learning models (Zhu et al., 2025). In another study, the GluLLM framework was proposed, which involved adapting a pre-trained LLM to the blood glucose prediction task through the use of a multimodal adapter. The results indicated that it outperformed 15 baseline models on an external validation dataset and was successfully deployed on a smartphone platform, thus demonstrating the feasibility of real-time on-device inference (Samadi et al., 2025).

Despite the encouraging progress described above, significant gaps remain in existing research. First, current CGM accuracy validation studies have focused primarily on patients with type 1 diabetes mellitus. However, the pilot population consists mainly of healthy or prediabetic individuals, whose blood glucose fluctuation characteristics differ fundamentally from those of patients with type 1 diabetes. Second, existing blood glucose prediction models have been trained and validated in ordinary daily-life settings, with little consideration being given to the impact of flight, a unique environmental factor, on metabolism. Mild hypoxia at high altitudes, psychological stress, and irregular eating and sleeping patterns can alter the body’s ability to regulate glucose levels. This means that models trained using ground-based data may experience reduced performance in aerial scenarios. Third, although multimodal fusion has been shown to improve predictive accuracy (Farahmand et al., 2025), the integrated variables in existing research are mainly daily-life variables such as insulin dosage, carbohydrate intake, and physical activity. There is a relative scarcity of studies that have incorporated physiological parameters such as heart rate and cerebral oxygen saturation into their model inputs. Fourth, the implementation of pilot blood glucose prediction systems still faces a series of practical challenges, including regulatory approval, model interpretability, and real-time inference efficiency.

To address the above issues, the present study aimed to develop a blood glucose prediction system for pilots based on CGM and an AI model. Its innovations include the following three aspects: (1) At the data level, CGM data were collected covering the entire flight process in a simulated flight environment. Multidimensional physiological parameters such as heart rate and cerebral oxygen saturation were simultaneously recorded to establish an aviation scenario-specific multimodal dataset. (2) At the model level, this study proposed a multimodal aviation scenario-specific transformer model, Aviation-GluFormer. It was designed with a dual-path attention mechanism that accounted for the physiological characteristics of blood glucose temporal data and the phase-specific features of aviation scenarios. Through a multimodal adaptation layer, it addressed the issues of heterogeneous feature fusion and enabled the accurate prediction of blood glucose fluctuations. (3) At the application level, the present study systematically assessed the timeliness of the prediction model in the early warning of hypoglycemic events and quantified the intervention time that AI-based predictions could provide pilots compared with that achieved with CGM monitoring alone. This provided empirical evidence to support future regulatory updates and clinical translation.

This study hypothesized that the Aviation-GluFormer model, which incorporates flight environment parameters, could accurately predict changes in the blood glucose levels of pilots within 15–60 min, with predictive performance superior to that of traditional methods. In addition, an early warning system based on this model could provide pilots with sufficient time to adopt intervention measures before the occurrence of hypoglycemia, effectively reducing the risk of in-flight incapacitation. Through this study, our objective was to provide a new intelligent tool for medical monitoring in aviation, allowing a paradigm shift from passive monitoring to active prevention in the management of pilots’ metabolic health.

## 2 Methods

### 2.1 Study subjects

This study was conducted at the Air Force Medical Center of the Chinese People’s Liberation Army from March 2025 to February 2026. *A priori* sample size estimation was conducted for this study, with the root mean square error (RMSE) of 30-minute blood glucose prediction as the primary outcome. Calculations were performed using G*Power 3.1: given an expected RMSE difference of 2.1 mg/dL between the proposed model and the LSTM baseline, a standard deviation of 3.8 mg/dL, a two-tailed α level of 0.05, and 80% statistical power, a minimum of 52 subjects was required. To meet the statistical power requirement and account for potential exclusion of invalid data, we ultimately enrolled 60 subjects. The inclusion criteria were as follows: (1) male; (2) aged 25–50 years; and (3) no history of serious illness within the previous year. Only male participants were enrolled in this initial study due to the limited size of our accessible volunteer cohort and the need to control for hormonal metabolic confounders in a small sample; we acknowledge this as a limitation of generalizability. The exclusion criteria included the following: (1) individuals diagnosed with diabetes or those currently taking antidiabetic medications; (2) individuals with other endocrine disorders that may affect glucose metabolism (e.g., thyroid dysfunction, Cushing’s syndrome); (3) individuals who had undergone surgery or had experienced severe trauma within the last three months; (4) individuals allergic to medical adhesives or with skin conditions causing an inability to use the sensor; or (5) individuals who were currently participating in other clinical trials.

All subjects were informed about the purpose, procedures, and potential risks of the study and provided written informed consent. The study protocol was reviewed and approved by the Medical Ethics Committee of the Air Force Medical Center of the Chinese People’s Liberation Army.

### 2.2 Research process and data collection

**Figure 1:**
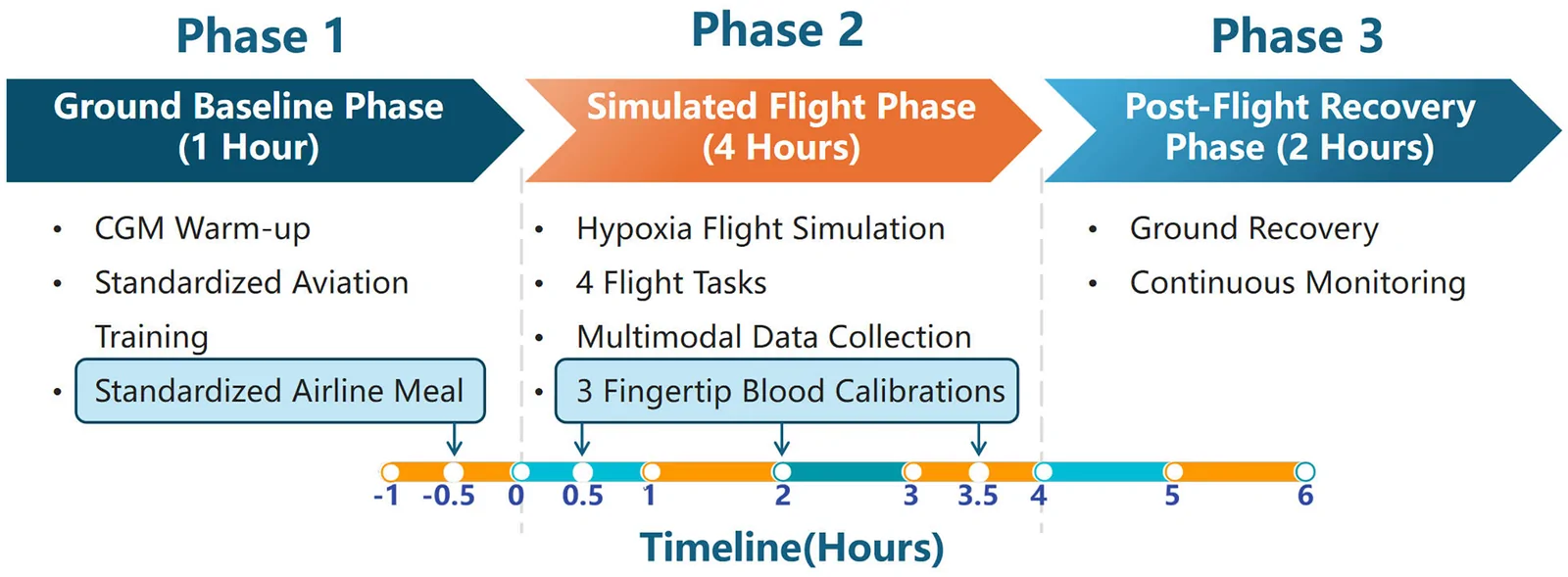
Experimental protocol of multimodal data acquisition in simulated flight environment for AI-driven glucose prediction.

Each subject was required to complete three simulated flight experiments, with at least a 1-week interval between the experiments to eliminate residual effects from the previous experiment and the influence of physiological cycles. The experimental process consisted of three phases, namely the ground (pre-flight) baseline phase, the simulated flight phase, and the post-flight recovery phase, which lasted for a total duration of 7 h (**Figure 1**).

Phase 1: Baseline phase on the ground (1 h). On the morning of the experiment, subjects arrived at the flight simulation laboratory on an empty stomach (having fasted for at least 10 h). First, the height and body weight of each subject were measured and the body mass index (BMI) was calculated. Subsequently, researchers applied a CGM sensor (Dexcom G7, Dexcom Inc., USA) to the posterior aspect of the upper arm on the non-dominant side of each subject. The sensor automatically recorded the interstitial glucose concentration every 5 min, which had a measurement range of 40–400 mg/dL and a manufacturer-validated MARD of 8.7%. After application of the CGM sensor, the subject rested in a seated position and watched a standardized flight training video while waiting for the device to warm up and stabilize. At 30 min into the baseline phase, the subject consumed a standardized airline meal (approximately 520 kcal, containing 60 g of carbohydrates, 25 g of protein, 20 g of fat, and 800 mg of sodium).

Phase 2: The simulated flight phase (4 h). At the end of the baseline phase, the subject entered a normobaric hypoxic chamber in which the oxygen concentration was reduced using nitrogen to simulate the typical commercial aircraft cruising environment. The oxygen concentration was adjusted to 15% (equivalent to an altitude of 2,400 m/8,000 ft), with a temperature of 22 ± 1 °C and humidity of 30 ± 5%.

**Figure 2:**
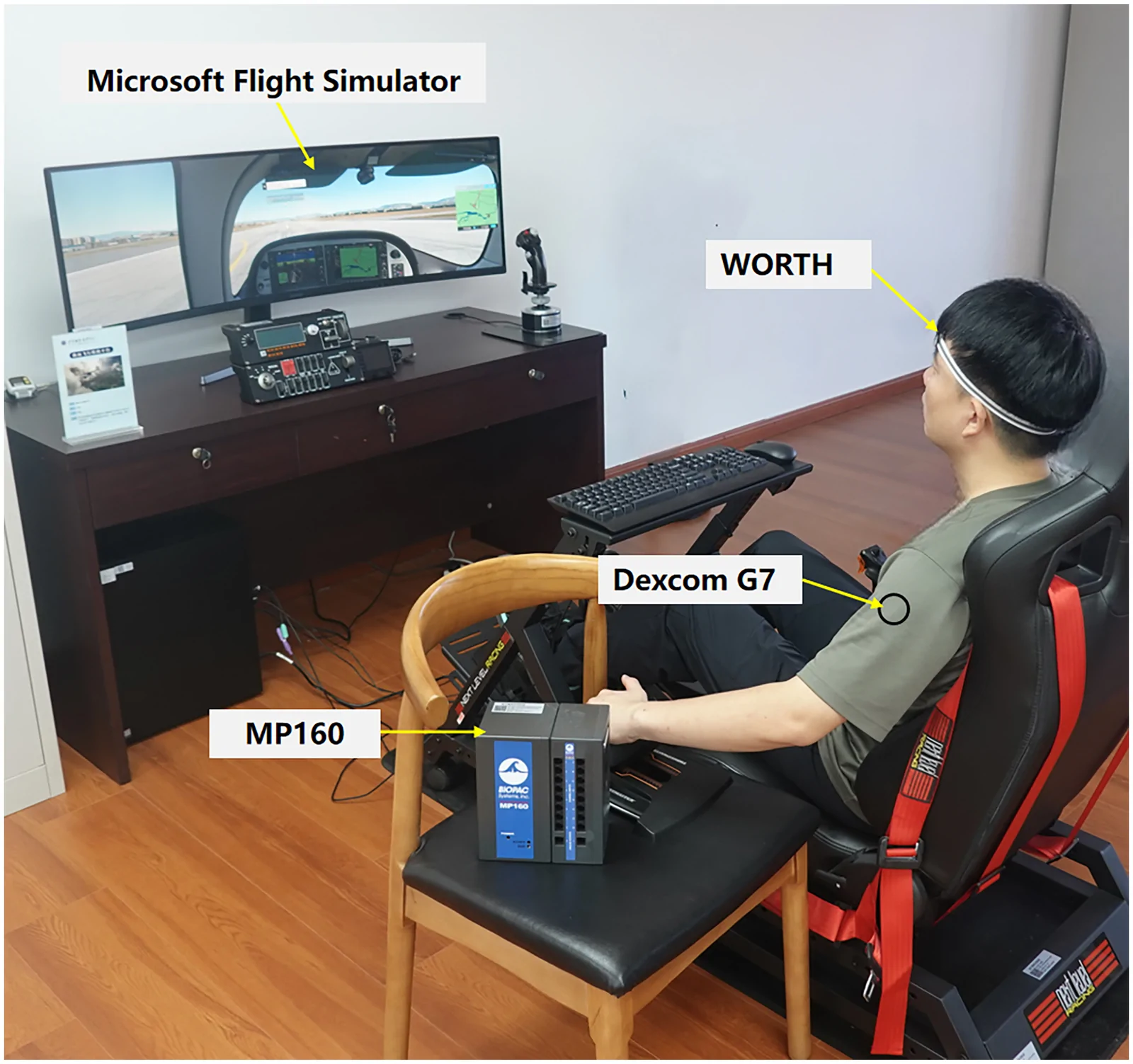
Schematic diagram of physiological monitoring and flight simulation scenario.

**Figure 2** shows the physiological monitoring and flight simulation scenarios. Regional cerebral oxygen saturation (rScO_2_) data were collected using a head-mounted cerebral oximeter (WORTH, Casibrain Technology Co., Ltd., China). Heart rate (HR) and three-dimensional (3D) acceleration during flight were measured with a multi-channel physiological data acquisition system (MP160, BIOPAC, USA), with the triaxial accelerometer secured to the subject’s non-dominant upper arm adjacent to the CGM sensor site. Physiological data were transmitted in real time through Bluetooth to an external monitoring workstation for recording.

The flight simulator featured a specialized seat (GT Track, Next Level Racing, Australia) and a 49-inch, 120 Hz curved display (LC49RG90SSCXXF, Samsung Group, South Korea). Microsoft Flight Simulator 2020 (Microsoft, USA) was adopted as flight simulation software, and control was performed using a universal flight stick set (T-16000M, Thrustmaster, France).

The flight task consisted of three phases, namely takeoff and climb, cruise, and descent and landing, with a total duration of 4 h. Thirty min after takeoff, the simulation entered the cruise phase. During this phase, adverse weather conditions, such as severe convective weather, blizzards, and thunderstorms, were introduced at random to increase the difficulty of the flight task and psychological workload. After 3.5 h, the simulation proceeded to the descent and landing phase. The combined effects of 4-hour continuous hypoxic exposure, high mental workload, and postprandial glucose consumption induced transient physiological hypoglycemia in some subjects during the middle-late cruise phase. All hypoglycemic events occurred spontaneously without pharmacological intervention, and subjects recovered spontaneously after the experiment without adverse reactions.

Throughout the flight task, the researchers monitored the subject’s status through cameras in the cabin and an intercom system and recorded the operational performance of the subject and the task phases. CGM data were continuously recorded. At key time points during the flight task (30 min after takeoff, mid-cruise, and 30 min before landing), fingertip blood samples were collected with a standard blood glucose meter (Contour Plus, Ascensia, Switzerland) for comparative validation to ensure the accuracy of CGM data.

Phase 3: Post-flight recovery phase (2 h). At the end of the simulated flight, the cabin pressure was restored to ground-level conditions and the subject rested in the recovery area after exiting the chamber. The CGM and physiological monitors continued to operate and record changes in blood glucose levels and physiological parameters during recovery. At the end of the recovery phase, all sensors were removed and a post-experiment interview with the subject was conducted.

### 2.3 Data preprocessing

All raw data were first exported in CSV format and aligned with a uniform timestamp. For CGM data, consecutive missing segments of 15 min or less were imputed by linear interpolation, whereas missing segments exceeding 15 min were excluded from the experimental data (no exclusions were required in this study).

Physiological monitoring data (HR, rScO_2_) and physical activity levels were averaged over 5-min intervals and aligned with the CGM time points. Physical activity level was computed as follows: first, the static gravity component was removed from each acceleration axis using a 2nd-order Butterworth high-pass filter (0.5 Hz cutoff frequency); the magnitude of the triaxial dynamic acceleration vector was then calculated, and the square root of the 5-min averaged magnitude was defined as the physical activity level. The final multimodal dataset included the timestamp for each time point and the corresponding CGM glucose level, HR, rScO_2_, physical activity level, and the flight task phase identifier (coded 1–4, which represented takeoff and climb, cruise, descent, landing, and recovery tasks, respectively).

All continuous features were standardized using Z-scores, and the mean and standard deviation were calculated solely based on data from the training dataset to prevent data leakage. Model input–output samples were established using the sliding window approach. The input window consisted of 60 min of historical data (12 time steps), and the prediction windows corresponded to glucose values at 15, 30, and 60 min in the future. The sliding window step size was set to 5 min to ensure that historical data were used to predict future values for all samples, without leakage of future information.

### 2.4 Establishment of prediction model: Aviation-GluFormer, a multimodal aviation scenario-specific transformer model

**Figure 3:**
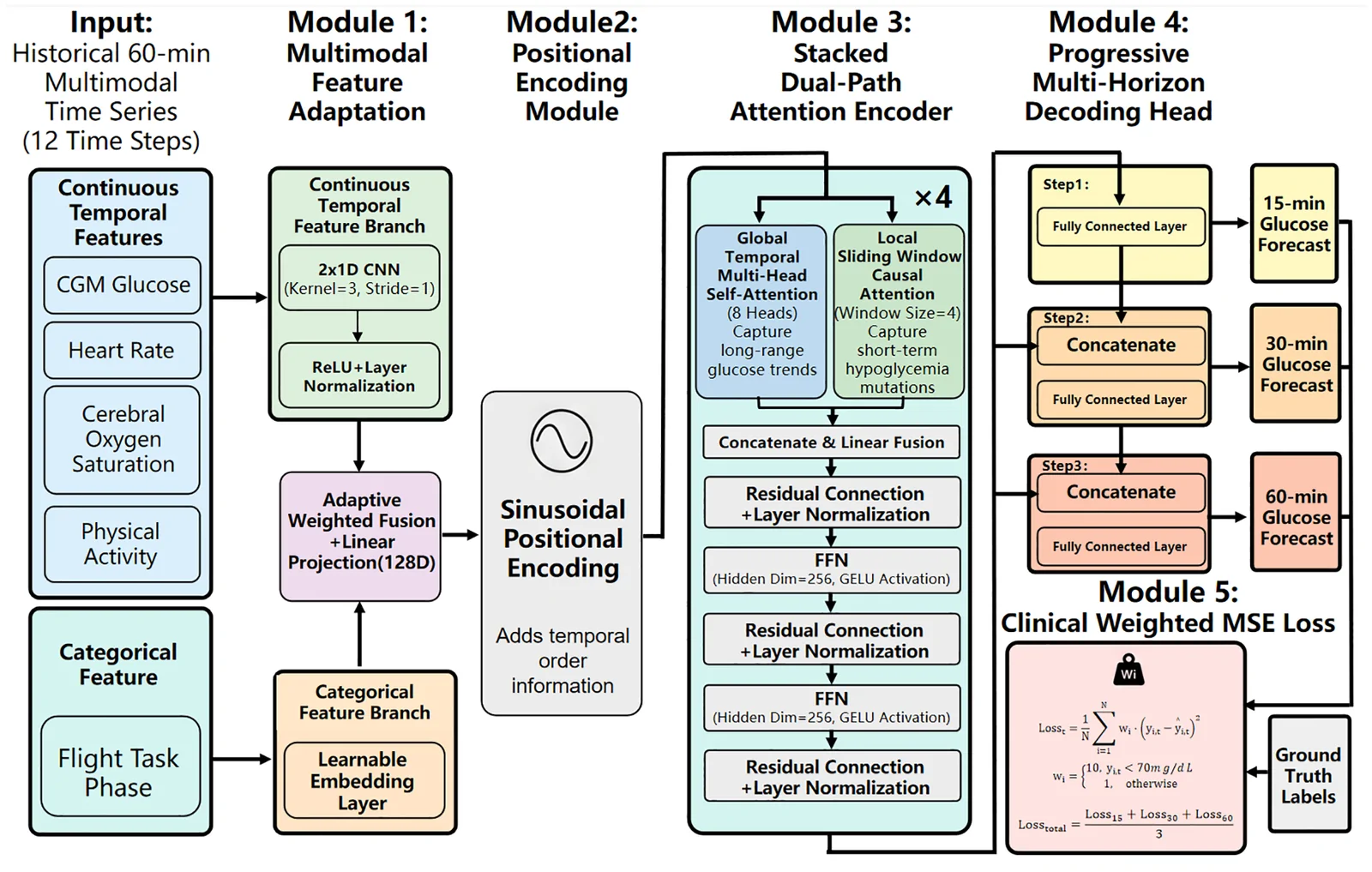
Overall architecture of the proposed Aviation-GluFormer model. The model consists of the following five core modules: multimodal feature adaptation layer, positional encoding module, progressive multi-horizon decoding head, dual-path attention encoder, and clinically weighted mean squared error (MSE) loss function. **Figure 4**. Training and validation loss curves of the model.

The Aviation-GluFormer multimodal temporal-aware transformer model was designed based on the specific requirements for predicting blood glucose level changes in aviation scenarios (long-range temporal dependencies, heterogeneous multimodal features, small-sample learning of hypoglycemia, and phase-specific metabolic patterns of flight). **Figure 3** shows the overall architecture of the model. The design of the core modules is shown in **Figure 3**.

To address the heterogeneity of input features, we designed a modality-specific embedding and alignment module to resolve the issue of differences in scale and distribution shifts across feature types.

For continuous temporal features such as CGM glucose level, HR, rScO_2_, and physical activity level, a two-layer one-dimensional convolutional neural network (CNN) was used to extract local temporal features. The size of the convolution kernel was set to 3, whereas the stride, padding, and number of output channels were 1, 1, and 64, respectively. The ReLU activation function and layer normalization (LN) were applied to capture short-term fluctuation patterns of each feature.

For categorical features, such as flight task phase identifiers, a trainable embedding layer was used for mapping to a 64-dimensional feature space, ensuring consistency with the continuous feature dimensions.

The features output from all branches were concatenated and mapped through a linear transformation into a 128-dimensional unified embedding space. To address the limitations of direct concatenation, which implicitly assumes equal contribution from all modalities, the learnable modality weight parameters were introduced. An independent trainable weighting coefficient was assigned to each modality, and the weights were automatically optimized through backpropagation during model training. This enabled an adaptive adjustment of the contribution of different physiological features to glucose prediction. The final output was a temporally aligned multimodal fusion embedding vector. This adaptation layer is designed to avoid the feature dilution problem of direct concatenation (a common limitation of standard multimodal Transformers) by adaptively adjusting the predictive weight of each physiological modality. Sinusoidal positional encoding was used to preserve temporal order information, with the encoding dimension matched to the feature embedding dimension (128 dimensions). The calculation formula used was the following:


$$PE_{\left(pos,2i\right)}=\sin\left(\frac{pos}{10000^{2i/d_{model}}}\right)$$

$$PE_{\left(pos,2i+1\right)}=\cos\left(\frac{pos}{10000^{2i/d_{model}}}\right)$$

where pos is the time step position (0–11, corresponding to historical time steps of 0–60 min), *i* is the dimension index (0–63), and *d_model_* = 128 is the feature embedding dimension. Intuitively, this sinusoidal encoding injects explicit temporal order information into the model, as the Transformer architecture has no inherent ability to recognize sequence order. It generates smooth, continuous temporal feature representations that allow the attention module to identify chronological glucose trends (such as gradual glucose decline during cruise phases) and capture time-lagged relationships between physiological signals (HR, rScO_2_) and subsequent glucose changes. Positional encoding was added in an element-wise manner to the multimodal fusion embedding vector and was fed into the subsequent encoder module.

Given the characteristics of blood glucose temporal data, a dual-path encoder was designed that combined global temporal self-attention with local sliding-window causal attention. The encoder consisted of four identical stacked encoder layers, with each layer possessing the following core structure:

A standard multi-head self-attention mechanism was employed, with eight attention heads and a dimension of 16 for each head. This was used to capture long-range dependencies across all time steps within the input window, which addressed the issue of information loss in long sequences commonly encountered in traditional RNN models.

We adopted a causal sliding-window attention mechanism with a window size of 4, which allowed attention to be directed only to the current time step and the preceding three historical time steps to strictly prevent the leakage of future information. This approach focused on capturing sudden trends and short-term dynamic characteristics of recent blood glucose levels, thereby improving the prediction accuracy of critical events such as hypoglycemia.

The attention outputs of the two paths were concatenated, fused via a linear transformation, and fed into a residual connection and LN layer. Subsequently, the output was passed through a two-layer feedforward network (FFN) with a hidden layer dimension of 256 and a GELU activation function. Finally, the output was fed through another residual connection and LN layer to complete the computation of a single encoder layer. This dual-path design balances global long-range glucose trend modeling (via full self-attention) and local abrupt hypoglycemia event capture (via sliding-window causal attention), addressing the weakness of standard single-path Transformers in detecting rare critical glucose drops in flight scenarios.

For multi-step prediction tasks with horizons of 15, 30, and 60 min, the traditional design involving parallel fully connected layers was abandoned, and a progressive decoding strategy was adopted that was better aligned with the physiological dynamics of blood glucose:

1. First, the hidden state of the last time step from the encoder output was fed into the first fully connected layer to generate the predicted blood glucose value at 15 min;
2. This 15-min prediction was concatenated with the hidden state output by the encoder and was subsequently fed into the second fully connected layer to produce the predicted blood glucose value at 30 min;
3. Similarly, the predicted result at 30 min was concatenated with the previous hidden state and fed into the third fully connected layer to generate the predicted blood glucose value at 60 min.

In this design, progressive prediction was employed to fully take advantage of the high-confidence results from the short-horizon predictions to optimize the long-horizon predictions. This reduced error accumulation and allowed for better alignment with the temporal progression patterns of blood glucose changes.

In view of the issues of limited hypoglycemia event samples and high aviation safety risk, and to accommodate the model’s synchronized three-horizon prediction (15, 30, and 60 min) output structure, a clinically weighted mean squared error (MSE) loss function was adopted in this study. The loss function supervised the predicted and actual values for the three prediction horizons, with the same loss computation method and the same supervision strength used for all horizons. Weighted differences between the different horizons were not established. The 10-fold weight for hypoglycemia samples was determined based on the inverse class frequency ratio, further adjusted to prioritize clinical safety and reduce missed detection of high-risk hypoglycemia events. For each prediction horizon t ∈ {15, 30, 60}, the loss was calculated using the following formula:


$${\text{Loss}}_{\text{t}}=\frac{1}{\text{N}}\sum_{\text{i}=1}^{\text{N}}{\text{w}}_{\text{i}}\cdot {\left({\text{y}}_{\text{i},\text{t}}-{\hat{\text{y}}}_{\text{i},\text{t}}\right)}^{2},\;{\text{w}}_{\text{i}}=\{\begin{array}{c} 10,\;{\text{y}}_{\text{i},\text{t}}<70\text{mg}/\text{dL}\;\\ 1,\text{ other blood glucose values} \end{array}$$

The total loss of the model was calculated by averaging the losses of the three prediction horizons:


$${\text{Loss}}_{\text{total}}=\frac{{\text{Loss}}_{15}+{\text{Loss}}_{30}+{\text{Loss}}_{60}}{3}$$

where N is the number of samples, y_i,t_ is the actual blood glucose value of the i-th sample at time t, ${\hat{y}}_{\text{i,t}}$ is the corresponding model-predicted value, and w_i_ is the clinical risk weight, which was applied only to hypoglycemia samples without distinguishing between prediction horizons.

### 2.5 Model training and validation

Data from all 180 flight experiments were randomly split into the training dataset (144 experiments), the validation dataset (18 experiments), and the test dataset (18 experiments) in an 8:1:1 ratio. This split ratio was selected to maximize the training sample size for our multimodal Transformer architecture, which requires sufficient data to learn dual-path attention parameters without underfitting, while retaining independent, equally sized validation and test sets for unbiased hyperparameter tuning and final performance evaluation. Supplementary sensitivity analysis using alternative 7:2:1 and 6:2:2 splits showed that smaller training proportions led to increased overfitting and higher hypoglycemia false negative rates, supporting the appropriateness of the 8:1:1 split for this dataset. During the splitting process, care was taken to ensure that data from the three experiments of the same subject were kept within the same dataset to completely prevent data leakage. The training dataset was used to update model parameters, the validation dataset was used for hyperparameter tuning and early stopping decision-making, and the test dataset was used for final performance evaluation.

The following settings were used:

Optimizer: AdamW (initial learning rate of 0.001, β_1_ = 0.9, β_2_ =0.999, weight decay 1e-4); learning rate scheduler: CosineAnnealingLR (cosine annealing, T_max = 50, minimum learning rate of 1e-6), batch size: 32, maximum number of training epochs: 100.

Early stopping strategy: Hyperparameter tuning was performed via grid search using only the validation dataset, with tuned parameters including initial learning rate (1e-4 to 5e-3), dropout rate (0.1 to 0.3), number of attention heads (4, 8, 12), and feed-forward hidden dimension (64, 128, 256). Early stopping was applied with a patience of 10 epochs: training was terminated if validation loss did not decrease for 10 consecutive epochs, and the model checkpoint with the lowest validation loss was selected as the final model. The validation dataset was used exclusively for hyperparameter tuning and early stopping, and did not influence the initial design of the model architecture. The test dataset was fully held out and only used for final, unbiased performance evaluation.

Dropout rate: 0.2 (applied to both the embedding and encoder layers to prevent overfitting); hardware environment: NVIDIA Tesla V100 32-GB GPU, CUDA 11.7, PyTorch 2.0.1 framework.

Training time: approximately 12 seconds per epoch; average stopping point: 42 epochs; total training time for a single model: approximately 8.4 min.

To systematically quantify the independent contribution of each input characteristic to blood glucose prediction performance and validate the effectiveness of the multimodal fusion strategy, we designed a progressive single-feature addition ablation experiment. In all ablation models, the same architecture, training hyperparameters, data split, and preprocessing workflow as the Aviation-GluFormer were adopted, with only the combination of input features altered to ensure a fair comparison. In the experiment, five variants of the progressive model were established, starting from a baseline model that included only the core blood glucose data. Only one additional feature was introduced at each step for the gradual construction of the full-modal model as follows:

Baseline model (only CGM): the input consisted solely of historical 60-min CGM glucose sequences without any other physiological or task features; CGM+HR model: established by introducing HR to the baseline model; CGM+HR+rScO_2_ model: established by adding rScO_2_ to the previous model; CGM+HR+rScO_2_+activity model: established by incorporating the physical activity level (3D acceleration magnitude) into the previous model; full-modal model: established by adding the flight task phase identifier to the previous model.

### 2.6 Comparison of baseline models

The superiority of the Aviation-GluFormer architecture was validated by comparing it with the following baseline models, with the data split, preprocessing workflow, and training strategy identical across all models. These baselines represent mainstream glucose prediction approaches with inherent limitations in aviation multimodal scenarios: linear extrapolation cannot capture non-linear physiological interactions; LSTM/GRU struggle with long-range dependencies across 60-min input windows; and the vanilla Transformer lacks modality-specific adaptation and targeted local critical-event capture, which our proposed architecture is designed to resolve.

1. Linear extrapolation: A linear trend was fitted using CGM data from the previous 30 min and was used to extrapolate future blood glucose values;
2. LSTM: A two-layer LSTM architecture was adopted, with a hidden layer dimension of 128, followed by a fully connected output layer. The input features were identical to those used in the full-modal model;
3. GRU: A two-layer GRU architecture was employed, with a hidden layer dimension of 128, followed by a fully connected output layer. The input features were identical to those of the full-modal model;
4. Standard transformer: A native transformer architecture consisting of a four-layer encoder and eight attention heads was used. The input features were identical to those of the full-modal model, but the multimodal adaptation layer and the dual-path attention design were omitted.

### 2.7 Evaluation of prediction performance for hypoglycemic events

A hypoglycemic event was defined as a CGM blood glucose value< 70 mg/dL. In the test dataset, we assessed the performance of the model in predicting hypoglycemic events that would occur within the next 30 min. A positive event was defined as three consecutive predicted blood glucose values of< 70 mg/dL. Sensitivity, specificity, positive predictive value (PPV), and negative predictive value (NPV) were calculated. The receiver operating characteristic (ROC) curves were plotted and the corresponding values of the area under the ROC curve (AUC) were calculated.

Clarke error grid analysis was used to assess the clinical safety of the model prediction results (Clarke et al., 1987). Using actual blood glucose levels measured by CGM as reference values (x-axis) and the model-predicted values as the test values (y-axis); the data points were divided into five clinical risk zones: A, B, C, D, and E. Zone A represented the clinically accurate zone, Zone B represented the benign error zone, Zone C represented the risk zone of overcorrection, Zone D represented the dangerous missed diagnosis zone, and Zone E represented the erroneous zone. The proportion of data points within each zone was calculated to evaluate the clinical accuracy and safety of the prediction results.

### 2.8 Analysis of the benefits of early-warning time

The exact onset time of hypoglycemia (glucose< 70 mg/dL) was determined via linear interpolation between two adjacent 5-minute sampling points where glucose crossed the 70 mg/dL threshold, to achieve finer temporal resolution than the raw sampling interval. When the predicted blood glucose level of the model was< 70 mg/dL for three consecutive 30-min predictions, the time point was recorded as the predicted onset time of a hypoglycemic event.

### 2.9 Statistical analysis

All evaluation metrics were selected to align with the specific goals of each analytical task, rather than using generic performance measures. For the continuous glucose prediction (regression) task, MAE, MAPE, RMSE, R² and Clarke Error Grid analysis were chosen: the first four quantify numerical prediction bias and variance explanation, while Clarke Error Grid assesses clinical safety—a core aviation medical requirement that standard regression metrics cannot capture. Mean absolute relative difference (MARD) was calculated to evaluate the agreement between CGM and fingertip blood glucose measurements, defined as the mean of |CGM glucose - reference glucose|/reference glucose × 100% across all paired samples. For the hypoglycemia event classification task, sensitivity, specificity, PPV, NPV and AUC were selected instead of overall classification accuracy. This is because hypoglycemia events represent a small minority of samples (severe class imbalance), and overall accuracy would yield misleadingly optimistic results by prioritizing the majority normal-glucose class, while the selected metrics better reflect the model’s ability to detect rare dangerous events and control false alarms.

Continuous variables were expressed as means ± standard deviation, whereas categorical variables were expressed as frequencies and percentages. Model performance metrics such as mean absolute error (MAE), mean absolute percentage error (MAPE), and RMSE were calculated by taking the average of prediction errors of each sample in the test dataset.

For comparison of per-sample prediction errors across models, linear mixed-effects models (LMMs) were used to account for repeated measures within participants. Model type was treated as a fixed effect, and participant identity was included as a random intercept to model inter-individual variability in prediction error. The variance explained by the participant random effect was reported to quantify participant-specific error variation. Four pre-specified pairwise comparisons were conducted between the Aviation-GluFormer model and each baseline model, with Bonferroni correction for multiple comparisons (adjusted significance threshold α’ = 0.0125).

One-way analysis of variance (ANOVA) was used to compare CGM data and fingertip blood samples. For hypoglycemia prediction performance, AUCs were compared using DeLong’s test, and model calibration was evaluated via the Hosmer-Lemeshow test (10 decile groups, df = 8), with p > 0.05 defined as the threshold for good calibration. A linear mixed-effects model with participant as a random intercept was used to compare predicted and measured hypoglycemia event onset times, to account for multiple events from the same participant. The difference between predicted and measured times was defined as the early warning lead time for hypoglycemia. All statistical analyses were performed using the SciPy and scikit-learn libraries in Python v.3.9, with the significance level set at α = 0.05 (two-tailed).

## 3 Results

### 3.1 Baseline characteristics of participants and overview of data

A total of 60 male volunteers were included in this study. The participants had a mean age of 37.6 ± 8.4 years (range: 25–49 years), a BMI of 24.3 ± 2.1 kg/m^2^, and an HbA1c level of 5.2 ± 0.4% (33 ± 2.1 mmol/mol). All subjects had HbA1c levels within the normal range (< 5.7%), thus meeting the study’s inclusion criteria.

Sixty subjects completed 180 valid simulated flight experiments, with no data points being excluded. A total of 17,280 CGM data points were collected (one data point every 5 min, which totaled 96 data points for each 8-h experiment) and a total of 540 fingerstick blood comparison measurements (three time points per experiment).

Comparison and validation of the CGM data and fingertip blood measurements revealed that the MARD was 9.7 ± 3.2% across all 540 paired data points. Stratified analysis by flight phase showed that MARD was 9.5 ± 3.0% at the cruise midpoint, 10.2 ± 3.5% at 30 min after takeoff, and 9.9 ± 3.3% at 30 min before descent. No statistically significant differences were observed among phases (one-way ANOVA: F = 1.87, p = 0.16).

### 3.2 Training process and model convergence analysis

**Figure 4:**
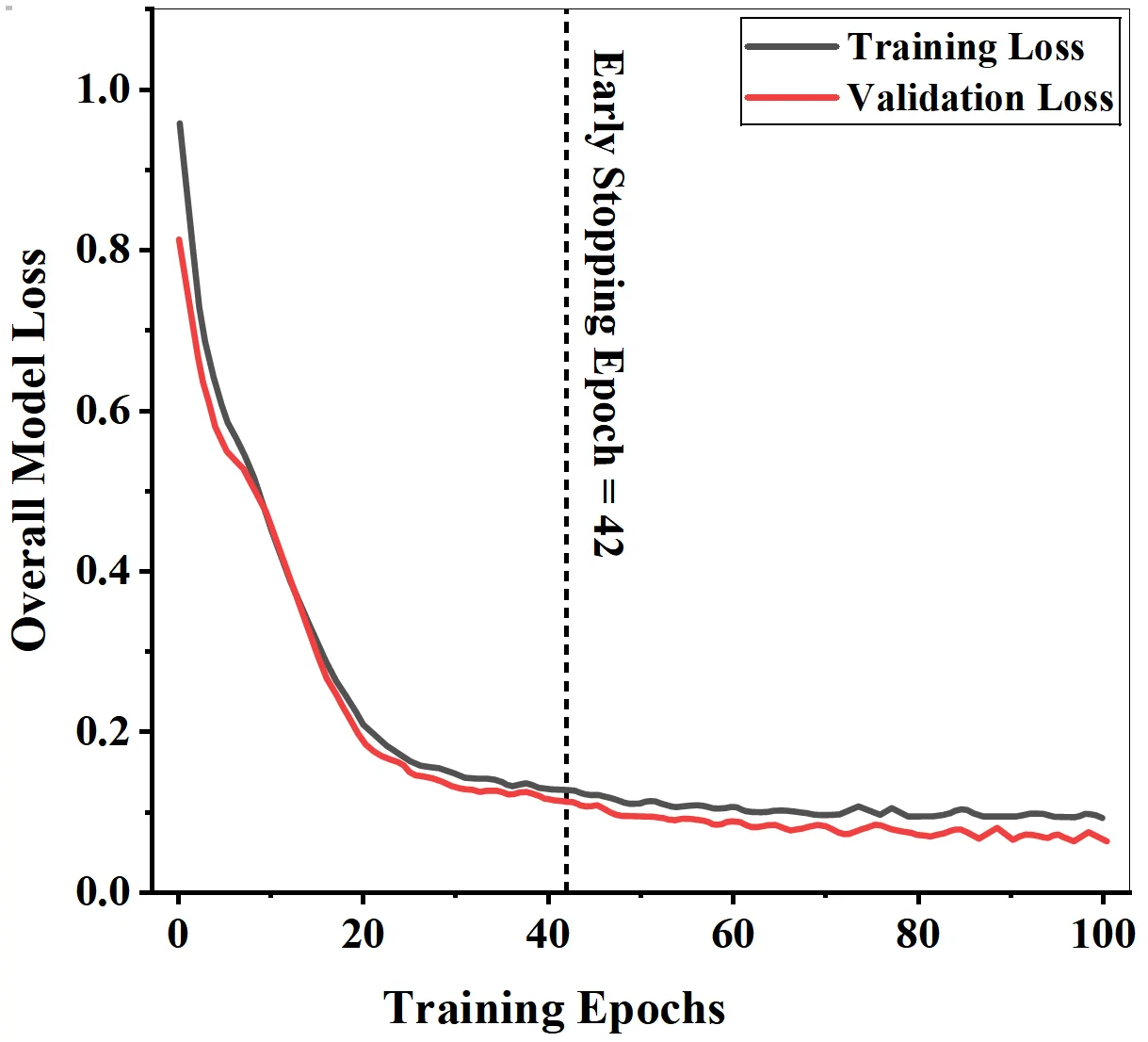
Training and validation loss curves of the model. The black line represents the training dataset loss, the red line represents the validation dataset loss, and the dashed line indicates the epoch at which early stopping was triggered (epoch 42).

**Figure 5:**
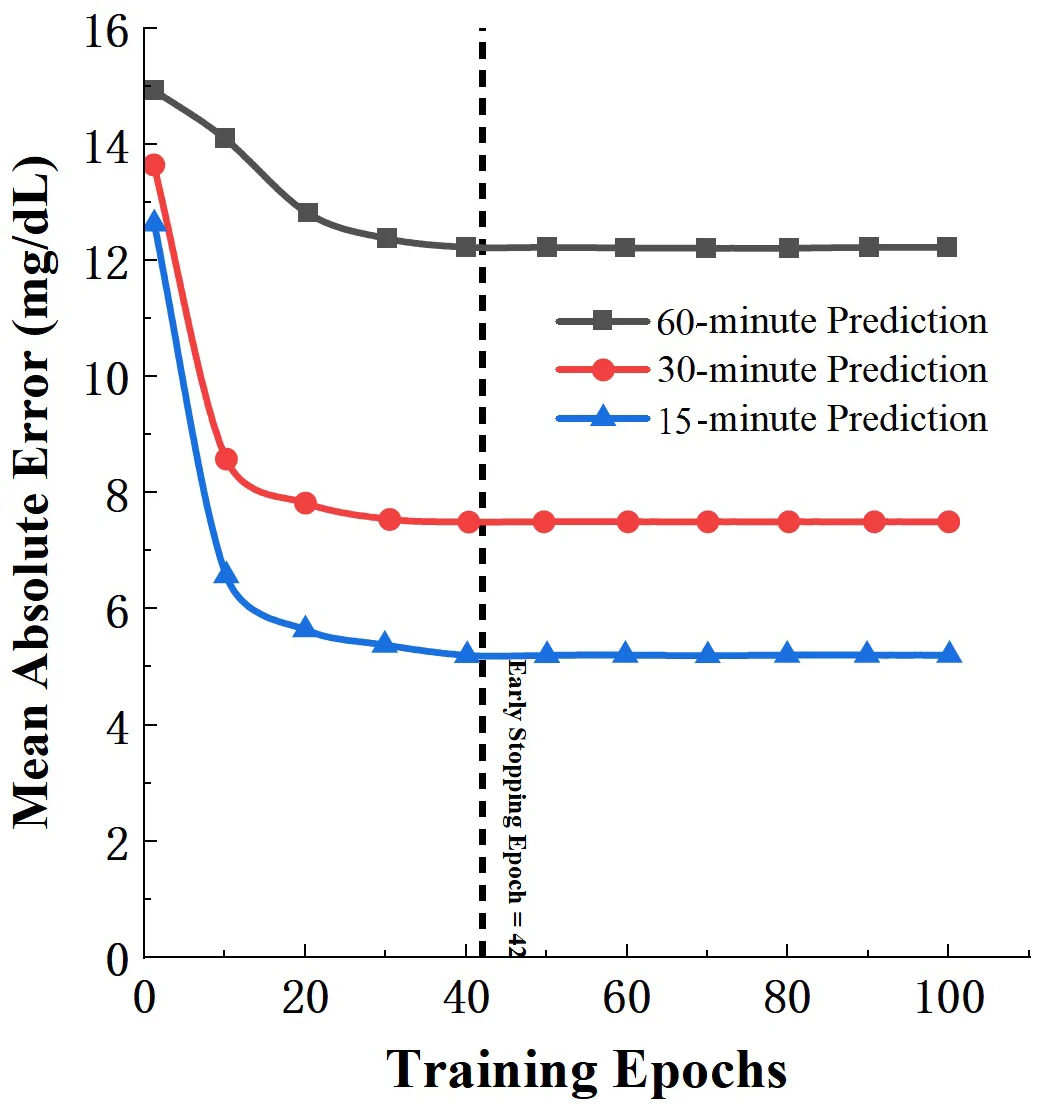
Validation MAE curves for different prediction horizons. Blue, red, and black lines indicate the 15-minute, 30-minute, and 60-minute prediction horizons, respectively.

**Figures 4**, **5** show the loss and performance curves during the model training process, based on the training and validation datasets. The loss of the training dataset decreased rapidly during the first 20 epochs and leveled off between 20 and 40 epochs. For the validation dataset, the loss decreased simultaneously with that of the training dataset, with no significant divergence. The early stopping strategy was activated at epoch 42, and the model did not exhibit overfitting.

The MAE of the validation dataset showed a trend consistent with the loss curve across training epochs. For the 30-min prediction horizon, the MAE of the validation dataset reached a minimum value of 7.4 mg/dL at epoch 42, which was consistent with the results of the test dataset. For the 15- and 60-min prediction horizons, the MAE of the validation dataset stabilized at 5.2 mg/dL and 12.3 mg/dL, respectively, indicating good model generalization performance.

### 3.3 Overall performance of the prediction model

**Table 1** shows the blood glucose prediction errors of the Aviation-GluFormer full-modal model across different prediction horizons on the independent test dataset (18 experiments with a total of 1,728 time points). For the 30-min prediction, MAE, MAPE, and RMSE were 7.4 ± 1.8 mg/dL, 7.2 ± 0.9%, and 9.4 ± 1.5 mg/dL, respectively. Prediction error increased as the prediction horizon lengthened, with the increase being gradual from 15 to 30 min but becoming more pronounced from 30 to 60 min.

**Table 1:** Prediction error of the model at different prediction horizons.

Prediction horizon	MAE (mg/dL)	MAPE (%)	RMSE (mg/dL)
15 minutes	5.2 ± 1.1 (4.62–5.73)	5.3 ± 0.7 (4.91–5.64)	6.8 ± 1.0 (6.31–7.32)
30 minutes	7.4 ± 1.8 (6.52–8.32)	7.2 ± 0.9 (6.73–7.61)	9.4 ± 1.5 (8.62–10.13)
60 minutes	12.3 ± 2.5 (11.04–13.55)	12.1 ± 1.6 (11.31–12.91)	15.6 ± 2.3 (14.48–16.77)

(data are expressed as mean ± standard deviation, with 95% bootstrap confidence intervals (1000 resamples), MAE, mean absolute error; MAPE, mean absolute percentage error; RMSE, root mean square error).

**Figure 6:**
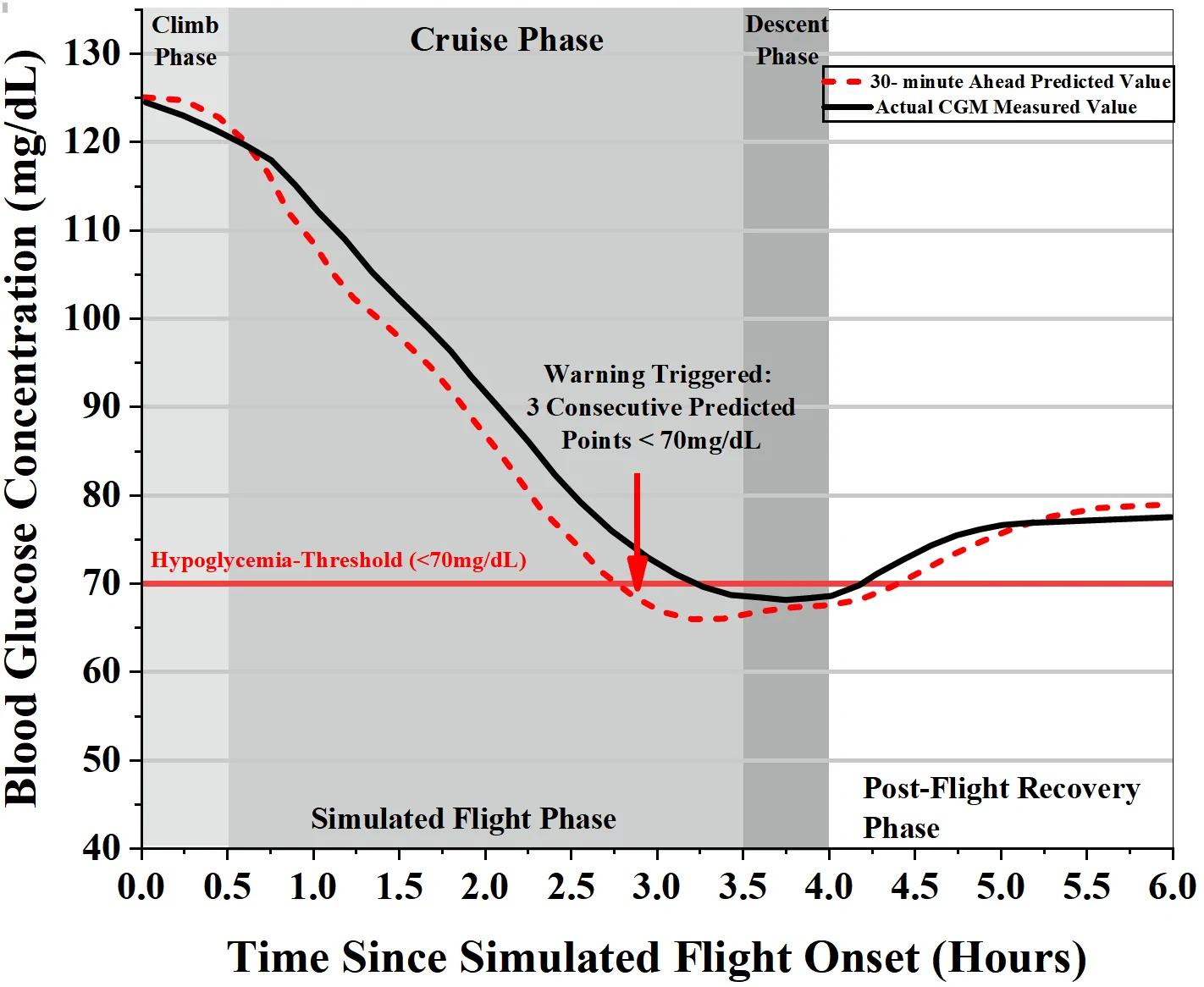
Blood glucose curve of a typical subject during a complete simulated flight and comparison with the model’s 30-minute predicted values. The solid black line represents the actual blood glucose levels measured by CGM, the red dashed line represents the model’s 30-minute predicted values, gray shaded areas denote the different flight phases (light gray = takeoff and climb, medium gray = cruise, dark gray = descent); the arrow indicates a warning signal.

**Figure 6** shows the blood glucose curve and the comparison of the measured values with the predicted values at 30-min predicted values of the model for a typical subject during a complete simulated flight process. It can be observed that the model accurately tracked the post-peak decrease in blood glucose levels and captured in advance the subtle hypoglycemic tendency that emerged during the cruise phase.

### 3.4 Performance comparison of different model architectures

**Table 2** shows the performance comparison among five different models for the prediction of 30-min blood glucose under the same data split. The Aviation-GluFormer full-modal model achieved the best performance. For the 30-min blood glucose prediction, the MAPE, RMSE, and R^2^ values were 7.2 ± 0.9%, 9.4 ± 1.5 mg/dL, and 0.91 ± 0.02, respectively, with RMSE lowered by 16.1% compared with the standard transformer model. All comparison results passed the Bonferroni-corrected significance test, confirming that the performance advantages of the proposed model were statistically significant. The participant random effect explained 18.3% of the total variance in prediction errors, indicating moderate inter-individual variability in model prediction accuracy.

**Table 2:** Performance comparison of different models for 30-minute glucose prediction.

Model	MAPE (%)	RMSE (mg/dL)	R^2^	*P*-value
Linear extrapolation	18.6 ± 2.3 (17.45–19.73)	22.3 ± 3.1 (20.76–23.84)	0.52 ± 0.06 (0.49–0.54)	<0.001
LSTM	10.4 ± 1.5 (9.64–11.15)	13.8 ± 2.2 (12.64–14.96)	0.81 ± 0.04 (0.79–0.83)	<0.001
GRU	10.8 ± 1.7 (9.95–11.65)	14.2 ± 2.4 (13.00–15.40)	0.79 ± 0.05 (0.76–0.82)	<0.001
Transformer	8.7 ± 1.2 (8.11–9.30)	11.2 ± 1.8 (10.31–12.12)	0.87 ± 0.03 (0.86–0.88)	<0.001
Aviation-GluFormer	7.2 ± 0.9 (6.76–7.65)	9.4 ± 1.5 (8.64–10.15)	0.91 ± 0.02 (0.90–0.92)	–

Data are expressed as mean ± standard deviation, with 95% bootstrap confidence intervals (1000 resamples). MAPE, mean absolute percentage error; RMSE, root mean square error; R², coefficient of determination. P-values are derived from linear mixed-effects models (with participant as a random intercept), comparing each baseline model against the Aviation-GluFormer model. Bonferroni correction was applied for 4 pairwise comparisons, with an adjusted significance threshold of α’ = 0.0125; all comparisons reached statistical significance.

### 3.5 Predictive performance for hypoglycemic events

**Figure 7:**
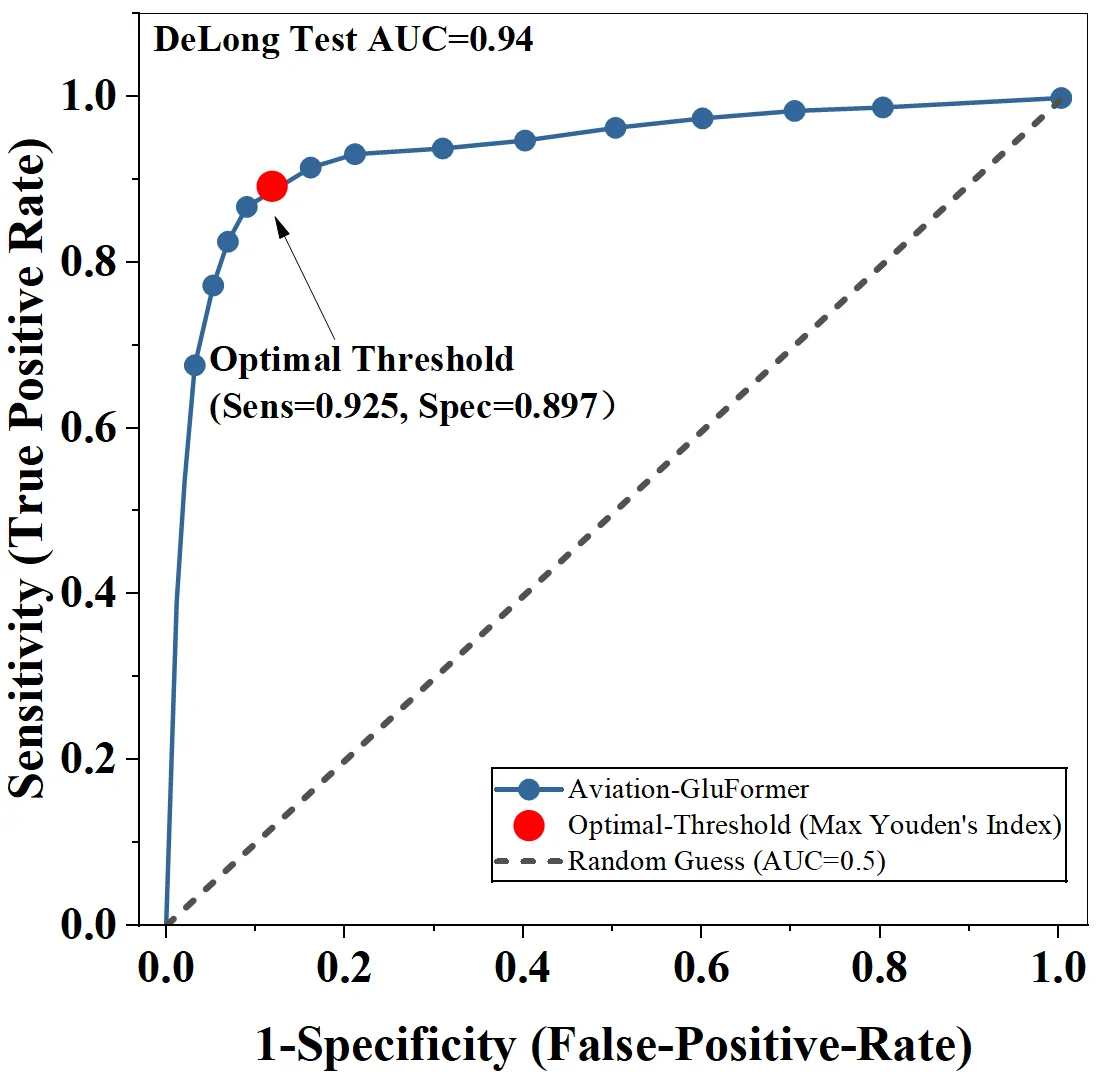
Receiver operating characteristic (ROC) curve for hypoglycemia event prediction. The red dot indicates the point with the highest Youden index (sensitivity = 0.925, specificity = 0.897), the diagonal line indicates the random guess line (AUC = 0.5).

In the training dataset (144 experiments), 37 hypoglycemic events were recorded; in the validation dataset (18 experiments), 5 hypoglycemic events were recorded. A total of nine hypoglycemic events occurred in the test dataset, corresponding to a hypoglycemia prevalence of 4.2% among all test time points, and all of these events took place during the second half of the cruise phase or the descent phase. As shown in **Figure 7**, the predictive performance of the model for hypoglycemic events within the next 30 min was as follows: sensitivity = 92.5%, specificity = 89.7%, PPV = 78.4%, NPV = 97.2%, AUC = 0.94 (95% confidence interval: 0.91–0.97).

**Figure 8:**
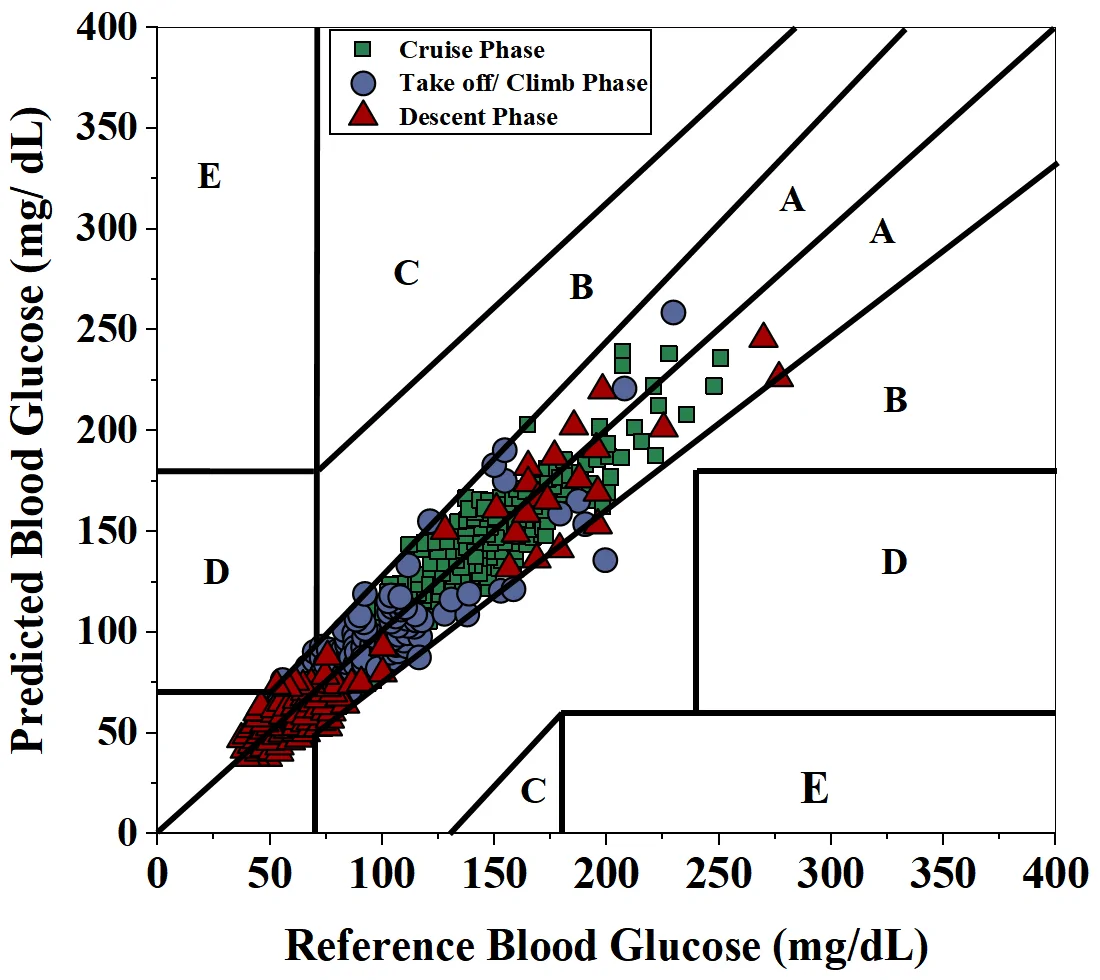
Clarke error grid analysis of model prediction results. Note: The horizontal axis represents actual blood glucose levels measured by CGM, and the vertical axis represents the model-predicted values. Colors of the data points indicate the flight phase (blue = takeoff and climb, green = cruise, red = descent). Zone A = clinically accurate zone, Zone B = benign error zone, Zone C = overcorrection risk zone, Zone D = dangerous missed diagnosis zone, Zone E = completely erroneous zone.

**Figure 8** shows the results of the Clarke error grid analysis. Among the 1,728 prediction points of the test dataset, 98.7% fell within the clinically accurate Zone A, 1.3% fell within the benign error Zone B, and none fell within Zones C, D, or E. All prediction points in the hypoglycemia range (< 70 mg/dL) fell within Zone A.

Model calibration was assessed via the Hosmer-Lemeshow test for hypoglycemia event prediction, which showed no statistically significant difference between predicted and observed hypoglycemia rates (χ² = 10.12, df = 8, p = 0.23), indicating good model calibration. 95% prediction intervals for continuous glucose predictions were estimated via bootstrap resampling; prediction uncertainty was higher for 60-min long-horizon predictions and glucose values outside the normal range, which is consistent with the expected error pattern of time-series prediction models.

### 3.6 Analysis of the benefits of early-warning time

All 9 hypoglycemic events in the test set were successfully predicted by the model. The average predicted onset time of hypoglycemic events was 253 ± 37 min, while the average measured onset time was 285 ± 34 min. Linear mixed-effects model analysis showed that the predicted onset time was significantly earlier than the measured time (P< 0.001), corresponding to an average early warning lead time of 32 ± 9 min. Sensitivity analysis using the Wilcoxon signed-rank test yielded consistent results (P< 0.001).

### 3.7 Results of the multimodal fusion ablation experiment

**Figure 9:**
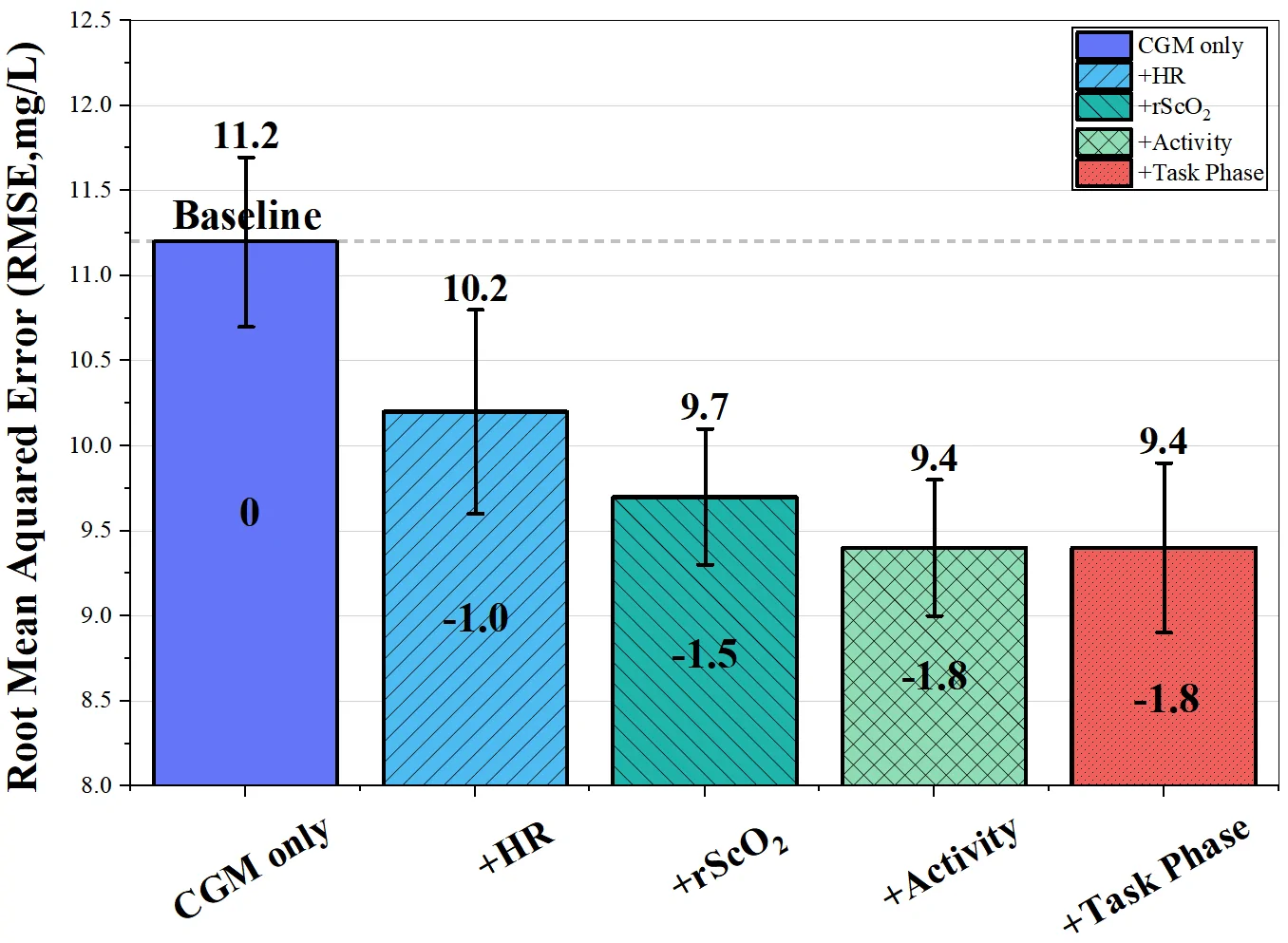
Prediction performance (RMSE) of models with different input feature combinations. “Baseline” refers to the CGM-only model; error bars represent the standard deviation; values above the bars indicate RMSE, while values within the bars indicate the reduction relative to the baseline.

**Figure 9** shows the predictive performance of the Aviation-GluFormer model with different combinations of input features. Compared with the baseline CGM-only model, HR provided the greatest contribution to performance enhancement (RMSE reduced by 1.0 mg/dL, a decrease of 8.9%), followed by rScO_2_ (RMSE reduced by 0.5 mg/dL, a decrease of 4.9%). The inclusion of physical activity level led to a slight performance increase, whereas the task phase identifiers did not improve performance. Paired t-tests with Bonferroni correction were performed to compare RMSE differences between sequential ablation models. The addition of HR significantly reduced prediction RMSE compared to the CGM-only baseline (p< 0.001), and the further addition of rScO_2_ also yielded a statistically significant RMSE reduction (p = 0.008). The incremental addition of physical activity level (p = 0.12) and flight task phase identifiers (p = 0.37) did not produce statistically significant performance improvements.

For hypoglycemia event prediction, the AUC showed a consistent upward trend with modality addition: the CGM-only baseline model achieved an AUC of 0.82; adding HR increased AUC to 0.89; further adding rScO_2_ increased AUC to 0.92; adding physical activity resulted in an AUC of 0.93; and the full model with flight phase identifier reached an AUC of 0.94. This indicates that multimodal physiological signals not only improve continuous glucose prediction accuracy but also enhance the discriminative ability for hypoglycemic events.

## 4 Discussion

### 4.1 Study findings

In the present study, an Aviation-GluFormer blood glucose prediction system was developed and customized to the specific operational scenarios of civil aviation pilots based on CGM and a multimodal transformer. The developed system was subsequently validated in a flight simulation environment. First, the overall MARD for CGM and fingertip blood was 9.7 ± 3.2%, with no statistically significant differences in MARD across the takeoff, cruise, and descent phases of flight. These results confirmed the stability of the Dexcom G7 device in a simulated aviation hypoxic environment and provided high-quality, scenario-specific baseline data for subsequent model training.

Second, as shown in **Figures 4**, **5**, the model training process demonstrated excellent convergence and generalization capabilities. The model triggered early stopping at epoch 42, with the training and validation dataset losses decreasing concurrently, and no significant overfitting was observed. The MAE of the validation dataset for prediction horizons of 15, 30, and 60 min stabilized at 5.2 mg/dL, 7.4 mg/dL, and 12.3 mg/dL, respectively. These results indicated that the early stopping strategy, dropout regularization, and clinically weighted loss function employed in this study effectively addressed the issues of small sample size and class imbalance in the aviation blood glucose dataset. This enabled the model to maintain stable performance even with a small-sample aviation blood glucose dataset, laying the foundation for future transfer learning in real flight data and deployment in onboard systems.

In terms of core predictive performance, the Aviation-GluFormer model exhibited differentiated accuracy characteristics across different prediction horizons (as shown in **Table 1**). The highest accuracy was achieved with 15-min prediction (MAE: 5.2 mg/dL, MAPE: 5.3%, RMSE: 6.8 mg/dL), whereas high stability was maintained with the 30-min prediction (MAE: 7.4 mg/dL, MAPE: 7.2%, RMSE: 9.4 mg/dL). However, prediction errors increased significantly for the 60-min horizon (MAE: 12.3 mg/dL, MAPE: 12.1%, RMSE: 15.6 mg/dL). Such patterns of change were fully consistent with the physiological dynamics of glucose metabolism: in the short term (15–30 min), blood glucose fluctuations were mainly influenced by prior food intake, basal metabolism, and immediate stress responses, resulting in clearer trends and higher predictability. In contrast, in the longer term (beyond 60 min), blood glucose was affected by multiple stochastic factors such as changes in flight task workload, compensatory responses to hypoxia, and autonomic nervous system regulation, leading to substantially increased uncertainty. Thus, the 30-min prediction horizon can achieve a balance between early warning lead time and prediction accuracy, making it an appropriate temporal window for real-time hypoglycemia alerting in pilots. The 15-min prediction can serve as a basis for precise confirmation following an alert trigger, whereas the 60-min prediction should only be used as a reference for long-term blood glucose trends and is not recommended for use in clinical decision-making.

As shown in **Table 2**, the Aviation-GluFormer model outperformed linear extrapolation and LSTM, GRU, and standard transformer models in terms of prediction accuracy in the 30-min blood glucose prediction task. This indicated that multimodal fusion and the dual-path attention mechanism effectively captured the temporal characteristics and physiological patterns of blood glucose fluctuations in an aviation environment. In terms of hypoglycemia detection (**Figure 7**), the model achieved a sensitivity of 92.5%, specificity of 89.7%, and AUC of 0.94. These results demonstrated that even in scenarios where mild hypoxia at high altitudes was compounded by flight stress, the model could still reliably identify the risk of impending hypoglycemia and lower the rate of both missed alerts and false alarms. Based on the analysis of the benefits of early-warning lead time, the model was capable of issuing hypoglycemia alerts 32 min earlier than real-time CGM, thus allowing pilots to complete their intervention before their blood glucose levels dropped to dangerous levels. Notably, the clinical alert rule requiring 3 consecutive positive 30-minute predictions to trigger an alarm will reduce the effective operational lead time. With a 5-minute prediction update interval, 3 consecutive confirmations take 10 minutes, resulting in an actual lead time of approximately 20 minutes before hypoglycemia onset. This window still allows sufficient time for in-flight interventions such as oral carbohydrate supplementation, and thus maintains clinical value for aviation safety.

As shown in **Figure 8**, the results of the Clarke error grid analysis indicated that 98.7% of the predicted points fell within Zone A, 1.3% within Zone B, and none within Zones C, D, or E. From a clinical perspective, this confirmed that the model’s predictions were safe and reliable and would not lead to incorrect treatment, excessive intervention, or dangerous missed diagnoses, thereby satisfying the high safety demands of aviation medicine. Even within the hypoglycemic range (<70 mg/dL), all predicted values fell within the clinically accurate range, further supporting the use of this system for proactive monitoring of hypoglycemia risk in pilots.

As shown in **Figure 9**, the results of the multimodal fusion ablation experiment indicated that the inclusion of HR, cerebral oxygen saturation, and physical activity level sequentially into the CGM-only model led to a continuous reduction in prediction error. This demonstrated that physiological signals during flight can provide effective incremental information for the prediction of blood glucose levels. HR provided the most significant contribution, indicating that sympathetic activation induced by flight stress precedes changes in blood glucose and serves as an important precursor for predicting blood glucose decline (Chen et al., 2024). The second largest contribution was provided by rScO_2_, which demonstrated the early influence of high-altitude hypoxia and cerebral energy metabolism on blood glucose fluctuations. Conversely, the flight task phase did not lead to a noticeable performance benefit. This suggested that its contextual information was already implicitly included within physiological signal changes, making it a redundant feature. The above results validated the rationality of the multimodal fusion strategy proposed in this study and can serve as a basis for feature selection in the development of lightweight models in future studies.

### 4.2 Innovative value and performance advantages of the Aviation-GluFormer Model

The Aviation-GluFormer model proposed in this study achieved a MAPE of 7.2% and RMSE of 9.4 mg/dL for the 30-min prediction horizon. Besides significantly outperforming traditional baseline models such as linear extrapolation, LSTM, and GRU, these results were also superior to those reported in previous studies of similar models. For instance, a transformer-based CGM-LSM model achieved an RMSE of 15.64 mg/dL for 1-h prediction horizon on the OhioT1DM dataset, while a DA-CMTL multi-task learning framework achieved an RMSE of 14.01 mg/dL for a 30-min prediction horizon (Luo et al., 2024; Hwang et al., 2025). The model’s performance advantages arose from the following four targeted design features that were better suited to the needs of blood glucose prediction in aviation scenarios:

The multimodal feature adaptation layer effectively addressed heterogeneous feature fusion: For different data types, including CGM data, physiological signals, and flight phase information, the design of modality-specific embedding, and adaptive weighting fusion mechanisms led to the avoidance of the feature dilution issue caused by traditional direct concatenation and enabled the extraction of predictive value from multimodal data.

The dual-path attention mechanism achieved a balance between long-range dependencies and the capture of local abrupt changes: The global attention design captured the long-range correlation between pre-prandial blood glucose levels and subsequent hypoglycemia, whereas the local sliding-window causal attention path focused on recent abrupt trends in blood glucose levels. This design addressed the limited capability of traditional transformers in capturing short-term critical events, thereby improving the accuracy of hypoglycemia event prediction.

Incremental decoding was aligned with the physiological dynamics of blood glucose: Instead of adopting a design of independent predictions through fully connected parallel layers, the present study employed progressive prediction, whereby high-confidence short-horizon predictions were leveraged to optimize longer-horizon predictions. This reduced error accumulation in long-horizon predictions and improved prediction stability.

The clinically weighted loss function was aligned with the core aviation safety requirements: By assigning a tenfold weight to high-risk hypoglycemia samples, the model addressed the class imbalance issue associated with learning from small samples of hypoglycemia events. Despite ensuring overall prediction accuracy, this strategy also enhanced the sensitivity of hypoglycemia event detection, thus avoiding missed detection of dangerous events.

In addition, excellent convergence and generalization capabilities were demonstrated in the model training process. The losses in the training and validation datasets decreased synchronously without signs of overfitting, and the training time for a single model was only about 8 min. These characteristics indicated the feasibility of edge-side deployment and real-time inference, thus laying a good technical foundation for future integration into onboard aviation systems.

### 4.3 Physiological mechanisms underlying the improvement of prediction performance through multimodal features

The results of the ablation experiment showed that HR and rScO_2_ were key auxiliary features for improving the prediction performance of the model. The inclusion of HR reduced RMSE by 8.9%, whereas the inclusion of rScO_2_ further reduced it by 4.9%. These results are supported by clear physiological mechanisms. Psychological stress during flight activates the sympathetic–adrenal medullary system, leading to increased release of catecholamines (epinephrine and norepinephrine). These hormones directly act on the liver to promote glycogenolysis and gluconeogenesis, while the intensity of stress is reflected by an increase in HR (Zhu et al., 2021). Therefore, changes in HR often precede fluctuations in blood glucose levels, thereby serving as an important precursor to changes in blood glucose. By capturing this temporal relationship, the model achieved more accurate early predictions, which were consistent with the findings of previous studies on the GlucoNet-MM framework (Luo et al., 2024; Hwang et al., 2025).

In the present study, rScO_2_ was selected for model training rather than conventional percutaneous arterial oxygen saturation (SpO_2_). This was due to the fact that rScO_2_ was more directly associated with the supply status of substrates involved in central cerebral energy metabolism in pilots. Since brain tissue lacks endogenous glycogen reserves, dynamic fluctuations in blood glucose directly affect cerebral glucose uptake efficiency and oxidative metabolism. rScO_2_ could detect subtle changes in central glucose metabolism at an earlier stage and could serve as a highly sensitive early-warning marker for blood glucose fluctuations in aviation scenarios. The ablation experiment in this study further confirmed that rScO_2_ effectively reduced the RMSE of the model predictions and provided additional predictive information. Under mild hypoxic flight conditions, the brain was the first organ to exhibit cerebral blood flow redistribution and compensatory responses, which disrupted glucose metabolic homeostasis. rScO_2_ was capable of capture these hypoxia-related metabolic disturbance patterns, making it particularly suitable for modeling in specialized aviation operational environments (Shaw et al., 2021). These findings extend prior ground-based research on HR- and SpO_2_-based glucose estimation to aviation scenarios, and further quantify the independent predictive value of cerebral oxygenation via ablation analysis (Mahmood et al., 2025).

In addition, high-intensity mental stress associated with flight preferentially triggers cerebral vasoconstriction and shifts in cerebral oxygenation rhythms. Since this physiological response occurred earlier than the drop in blood glucose levels, it provided the model with longer-term leading indicators, effectively extending the safety warning window for hypoglycemia (Chen et al., 2024; Demir et al., 2026).

### 4.4 Limitations of the study and future prospects

Despite the promising findings outlined in this study, certain limitations must be acknowledged. First, all data were collected in a simulated flight environment within a normobaric hypoxic chamber. Factors such as vibrations, radiation, extreme temperature fluctuations, and real-time task loads encountered during actual flights may affect the accuracy of CGM devices and the generalizability of the model. Therefore, further research using real flights is required to validate the performance of the model. Second, the study sample size was relatively small and all subjects were male. Given established sex differences in glucose metabolism and sympathetic stress response to hypoxia, caution must be exercised when extrapolating the study’s findings to female pilots and general aviation populations. Follow-up real-flight validation studies will enroll a balanced sex cohort to evaluate cross-sex generalizability and optimize sex-adaptive feature weights. Third, the study sample was limited to metabolically healthy male pilots, and the model is not validated for pilots with diabetes or prediabetes. Glucose regulatory mechanisms differ fundamentally between healthy individuals and individuals with diabetes: healthy pilots maintain glucose homeostasis through intact endogenous hormonal regulation, with fluctuations mainly driven by environmental stress and task load; while pilots with diabetes rely on exogenous insulin intervention, with more complex and larger-amplitude glucose fluctuations. In future work, we will explore transfer learning and domain adaptation strategies to extend the model to diabetic pilot populations based on small-sample real-world data. Fourth, the number of hypoglycemic events in the test set was limited (9 events), which may result in relatively wide confidence intervals for point estimates of sensitivity and AUC, and the robustness of event-level prediction performance needs to be further verified in larger cohorts. Fifth, although the Aviation-GluFormer model achieved an improvement in interpretability through its dual-path attention design, the “black box” nature of deep learning models may still pose obstacles to regulatory approval and clinical application. In subsequent studies, the clinical interpretability of the model should be improved by employing methods such as attention weight visualization and the use of large language models to generate natural-language explanations for predictions.

In future studies, expansion of both the sample size and the scope of the study population will be required. Notably, current findings are only generalizable to metabolically healthy, medically screened commercial pilot populations; results cannot be directly extrapolated to individuals with diabetes, prediabetes, or non-aviation general populations, and further cross-cohort transfer validation is needed.

In terms of extended application, the multimodal glucose prediction framework proposed in this study also has potential application value in fully automated artificial pancreas systems for diabetic pilots. As an independent safety warning module, the model can integrate multi-dimensional physiological signals such as heart rate, cerebral oxygen saturation and physical activity to detect impending hypoglycemia earlier than CGM monitoring alone, and trigger the artificial pancreas system to suspend insulin infusion or prompt carbohydrate supplementation in advance, so as to reduce the risk of severe hypoglycemia in special operational scenarios (Askari et al., 2023; Ahmadasas et al., 2025). The dual-path attention mechanism and multimodal adaptation layer designed in this study can also provide methodological reference for the optimization of artificial pancreas systems under complex environmental stress such as aviation hypoxic conditions, helping to improve the safety and robustness of automated insulin delivery in special populations and scenarios.

## 5 Conclusions

In conclusion, the present study developed and validated a multimodal transformer-based blood glucose prediction system tailored for aviation scenarios. The system can accurately predict blood glucose changes in pilots over 15–60 min based on CGM data and can provide highly sensitive early warnings of hypoglycemic events. It offers high prediction accuracy for the 30-min prediction horizon, has good clinical safety, and delivers timely early warnings. By transforming traditional passive blood glucose monitoring into proactive risk alerting, the system serves as a new intelligent approach to ensuring flight safety.

## Data Availability

The raw data supporting the conclusions of this article will be made available by the authors, without undue reservation.
